# A retrospective, comparative, clinical study of occlusion rate of peripherally inserted central catheters fabricated of poly(vinyl alcohol)-based hydrogel composite

**DOI:** 10.1007/s10856-023-06736-0

**Published:** 2023-07-21

**Authors:** Joseph Bunch, Brian Hanley, Daniel Donahue

**Affiliations:** 1 ProVasc Solutions, LTD. Vascular Access Group, Romeoville, IL USA; 2 Access Vascular Inc., Billerica, MA USA

## Abstract

Thrombotic accumulation is associated with surface interactions between blood proteins and vascular access devices. Catheter occlusion results from this process, and is a costly, common, occurrence with peripherally inserted central catheters (PICCs). Hydrophilic catheter materials exhibit antithrombotic properties. This retrospective study evaluates the occurrence of catheter occlusion of PICCs constructed of a poly(vinyl alcohol)-based hydrogel composite known as hydrophilic biomaterial (HBM), compared to thermoplastic polyurethane (TPU) control devices. A total of 121 PICCs, 60 HBM and 61 TPU, were placed in patients with a clinical need and were reviewed for the occurrence of catheter occlusion. The records review found that occlusions occurred in 0/60 (0.0%) of the HBM PICCs and 13/61 (21.3%) of TPU PICCs (*p* = 0.001). HBM exhibits favorable qualities for vascular access, most importantly its extreme hydrophilicity. Clinically, this may be responsible for the reduction in PICC occlusions, which could improve patient outcomes.

**Graphical Abstract:**

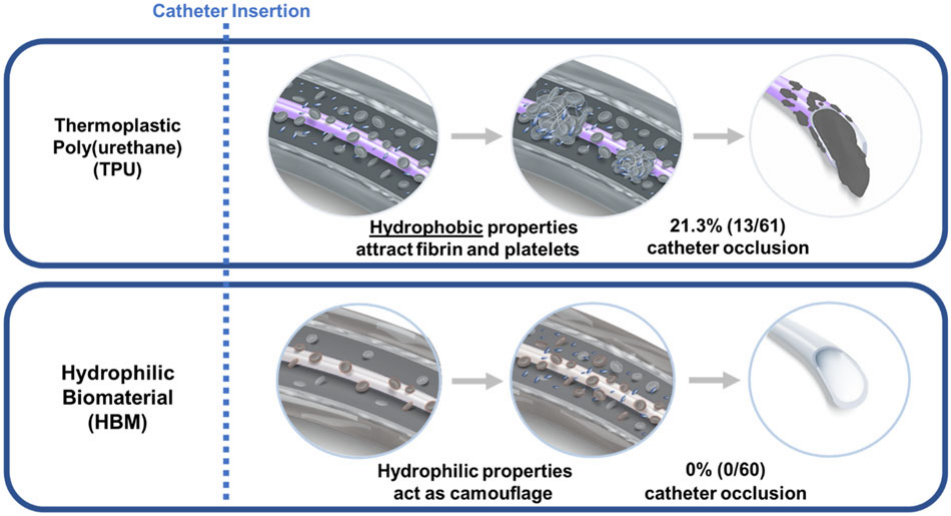

## Introduction

One of the most desirable qualities of biomaterials used for blood-contacting medical device applications is low thrombogenicity, especially in the field of vascular access. Roughly 60–90% of all admitted hospital patients receive a venous access device (VAD) during their stay [1], and over 2.7 million peripherally inserted central catheters (PICCs) per year are inserted in the USA alone [2]. For the subset of patients that require treatment to be administered in a central vein, a PICC may likely be selected as the appropriate choice. Reasons may include that venous access required for more than 14 days, infusion of certain types of medications requiring central venous access, or lack of suitable access in peripheral veins [3]. Introduction of a PICC into the vein activates a foreign body response that culminates in thrombus formation [4]. As a result, thrombotic PICC occlusions are a commonly reported adverse event and often lead to complications causing delays in treatment, increased cost, and removal [5, 6].

When a VAD is inserted, blood proteins (such as fibrinogen and collagen) and host cells (such as platelets) begin to attach to the surface of the device. This starts the formation of adherent material in and around the catheter surface. The magnitude and rate of the biological response is dependent upon the materials used in the construction of the catheter as well as the effect of shape/design affecting blood flow [7]. Poor protein resistance is often associated with surface hydrophobicity, which generates a high surface energy that the body relieves via protein adsorption [8]. Proteins undergo a conformational change to associate their hydrophobic domains with the biomaterial surface and their hydrophilic domains with the biological environment to create a substantial reduction in surface energy [9].

The most commonly used material to construct VADs is thermoplastic polyurethane (TPU). Medical grade TPU polymers have been well studied over 30 years of use in vascular access applications and have been proven to be safe and effective. However, it remains predominantly hydrophobic and therefore susceptible to non-specific protein adsorption [10, 11]. A less commonly used material to construct VADs is hydrogel. Hydrogels are inherently hydrophilic and have been used in many medical device applications to increase the biocompatibility of implanted devices [12]. A challenge with using hydrogels to construct a VAD is a lack of mechanical strength. Access Vascular Inc (AVI) has synthesized a novel hydrogel composite biomaterial that has been dialed in to possess the strength and durability required to be used as a VAD, hereafter referred to as hydrophilic biomaterial (HBM). Unlike a surface coating, this material constitutes the entire catheter lumen of the catheter. PICCs and midline catheters (MCs) constructed of HBM have been cleared by the United States Food and Drug Administration [13, 14] and are commercially available; however, there is limited evidence demonstrating the impact of these catheters on thrombosis clinically. This retrospective clinical study investigates whether PICCs constructed of HBM may reduce thrombotic catheter occlusions compared to the standard of care, TPU devices, in clinical practice.

## Materials and methods

### Study design

This was a retrospective clinical study of PICC lines inserted in 121 subjects between August 2020 to October 2020. Sixty-one subjects received a standard of care control device, a 4Fr Single Lumen PICC constructed of TPU, and 60 subjects received a HBM device (HydroPICC® 4Fr Single Lumen; Access Vascular Inc., Billerica, MA, USA) as part of their medical care. The study protocol was reviewed by Pearl Institutional Review Board (Indiana, USA), reference number 20-ACCE-101. It was determined to be exempt according to US FDA CFR 56.104 and 45CFR46.104(b)(4): (4) secondary research uses of data or specimens on November 10, 2020.

The PICC insertions were performed at either an acute care hospital or a long-term acute care hospital serviced by ProVasc, Ltd. vascular access specialists in the Chicagoland area (Illinois, USA). All contracted inserters were registered nurses specifically trained in the insertion of PICCs utilizing maximal sterile barrier precautions and real-time ultrasound guidance. Per the policies of the vascular access service provider, the clinician was required to verify that the choice of VAD was appropriate based on the patient’s history and the recommendations provided in the latest standards published by the Infusion Nurses Society (INS) [3]. Follow-up care and PICC maintenance were performed per each facility’s standard operating procedures, compliant with the latest INS standards [3]. Data were gathered by qualified vascular access clinicians from the vascular access team in a standardized case report form.

Eligible records were those of patients ≥18 years of age at the time of insertion who received a PICC constructed of HBM or TPU that was placed by the contracted vascular access service. Records were excluded if the patient was on dialysis or received a PICC constructed of materials other than those studied, such as silicone. Patients were situationally randomized by receiving the specific type of PICC contracted for use at each facility. Since there was a much larger volume of patients that received TPU PICCs during the timeframe, for each HBM subject eligible for the study, a TPU subject was selected with a similar date and time of insertion until at least 60 of each were identified.

Demographics collected included gender, age, BMI, race, ethnicity, and insertion characteristics included arm inserted, vein accessed, arm circumference, number of attempts, and reason for PICC. Occlusions requiring intervention, with either resolution with a thrombolytic agent or removal/replacement, were also collected. Catheter occlusions were defined as the inability to infuse or aspirate through the device during catheter maintenance or use.

All analysis was performed according to the material group. Data were summarized using descriptive statistics by group or for all subjects combined. Descriptive statistics for numeric variables include the number of observations (*n*), mean, and standard deviation. Categorical variables were summarized using counts and percentages, where percentages were calculated based on the total subjects or available data. Statistical methods were used to compare the treatment groups and a two-sided *p* value less than or equal to 0.05 was considered statistically significant. For numeric variables, the Wilcoxon rank-sum test was used to evaluate if the groups had a similar location (e.g., median). For categorical outcomes, Fisher’s exact test was used to evaluate the null hypothesis that the levels of the categorical variable were similarly distributed across the groups. The primary endpoint, the incidence of catheter occlusions, was analyzed with a Fisher’s exact test. Another statistical testing was performed to assess the homogeneity of baseline characteristics of the two groups. A total sample size of 60 per group was selected to provide at least 80% power under the assumption the difference in the success rates is at least 26% based on a normal approximation.

### HBM

HBM is a composite of hydrophilic polymers and is a bulk material making up the entire catheter body, not a hydrophilic coating. HMB is created using multiple hydration and dehydration steps with various solutions, temperatures, and pressures to extrude it for use in catheters. The monomer consists primarily of PVA that is impregnated with poly(acrylic acid). It is heat treated and physically crosslinked (hydrogen bonding) to create a high-strength hydrogel material; no chemical cross-linking agents are used [15]. The molecular weight of HBM is in the range of its PVA base ~100,000 to ~200,000 g/mol, and near the average MW of the non-crosslinked PVA polymer, ~150,000 g/mol. Access Vascular’s HydroPICC and HydroMID devices are FDA cleared which confirms that biocompatibility testing has been successfully completed according to ISO 10993 for externally communicating devices contacting blood. The contact duration is considered permanent (>30 days) and the contact duration prolonged (24 h to 30 days) or long term (exceed 30 days) depending on the type of VAD [13, 14].

## Results

The total number of records reviewed was 121, 60 in the HBM group and 61 in the TPU group. Demographic and insertion characteristic data showed no significant statistical differences within the two groups, including medication type infused (Tables 1 and 2). The occurrence of occlusion in the HBM group was dramatically lower than in the TPU group. Of the 60 PICC insertions in the HBM group, no occlusions were reported. In the TPU group, 13/61 (21.3%) catheter occlusions were reported (*p* = 0.001). Five of the thirteen catheter occlusions were resolved with a thrombolytic agent and eight PICCs were replaced due to loss of function of the PICC secondary to occlusion (Table 3).

**Table 1:** Demographics of the study population

	Total *N* = 121	HBM group *N* = 60	TPU group *N* = 61	*p* value
Gender				
Male (%)	53 (43.8)	27 (45.0)	23 (38.3)	0.251^a^
Female (%)	58 (47.9)	30 (50.0)	31 (51.7)	
Missing (%)	10 (8.3)	3 (5.0)	7 (11.5)	
Age (years)				
Mean ± SD	72 ± 14.2	74 ± 14.3	69 ± 13.8	0.059^b^
BMI (kg/m^2^)				
Mean ± SD	29 ± 8.4	30 ± 8.4	29 ± 7.9	0.414^b^
Race				
Black/African American (%)	20 (16.5)	8 (13.3)	12 (19.7)	0.153^a^
White (%)	89 (73.6)	49 (81.7)	40 (65.6)	
Not reported (%)	12 (9.9)	3 (5.0)	9 (14.7)	
Ethnicity				
Hispanic or Latino (%)	5 (4.1)	2 (3.3)	3 (4.9)	0.059^a^
Non-Hispanic or Latino (%)	101 (83.5)	55 (91.7)	46 (75.4)	
Not reported (%)	15 (12.4)	3 (5.0)	12 (19.7)	

*BMI* body mass index, *HBM* hydrophilic biomaterial, *SD* standard deviation, *TPU* thermoplastic polyurethane

^a^Fisher’s exact test

^b^Wilcoxon rank-sum test

**Table 2:** PICC line insertion characteristics

	Total *N* = 121	HBM group *N* = 60	TPU group *N* = 61	*p* value
Arm inserted				
Left (%)	42 (34.7)	25 (41.7)	17 (27.9)	0.129^a^
Right (%)	79 (65.3)	35 (58.3)	44 (72.1)	
Vein accessed				
Basilic (%)	92 (76.0)	47 (78.3)	45 (73.8)	0.868^a^
Brachial (%)	25 (20.7)	11 (18.3)	14 (22.9)	
Cephalic (%)	4 (0.3)	2 (3.3)	2 (3.3)	
Number of attempts				
1 (%)	115 (95.0)	57 (95.0)	58 (95.0)	1.000^a^
2 (%)	6 (5.0)	3 (5.0)	3 (5.0)	
Reason for PICC				
Antibiotics (%)	115 (95.1)	55 (91.6)	60 (98.4)	0.130^a^
Blood/fluids (%)	4 (3.3)	3 (5.0)	1 (1.6)	
TPN (%)	1 (0.8)	1 (1.7)	0 (0.0)	
Missing (%)	1 (0.8	1 (1.7)	0 (0.0)	

*HBM* hydrophilic biomaterial, *PICC* peripherally inserted central catheter, *SD* standard deviation, *TPN* total parenteral nutrition, *TPU* thermoplastic polyurethane

^a^Fisher’s exact test

**Table 3:** Summary of catheter occlusions

	HBM group *N* = 60	TPU group *N* = 61	*p* value
Total occlusions (%)	0 (0.0)	13 (21.3)	0.001^a^
Occlusion-related replacements (%)	0 (0.0)	8 (13.1)	0.006^a^
Occlusions resolved with thrombolytic agent (%)	0 (0.0)	5 (8.2)	0.057^a^

*HBM* hydrophilic biomaterial, *TPU* thermoplastic polyurethane

^a^Fisher’s exact test

## Discussion

The goal of a vascular access professional is to minimize complications by considering each patient’s unique situation [16]. Proven ways to accomplish this are by choosing the best VAD for the prescribed care, improved insertion techniques, and better maintenance routines [17]. This research explored whether choosing the appropriate catheter material may be another effective way to further reduce complications.

**Fig. 1:**
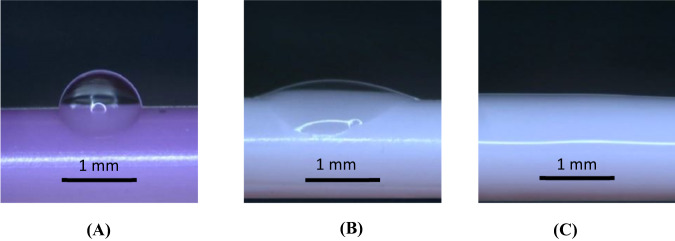
Optical images of 2 µL water droplets on **A** thermoplastic polyurethane catheter, **B** HBM dehydrated catheter, and **C** HBM hydrated catheter [25]

**Fig. 2:**
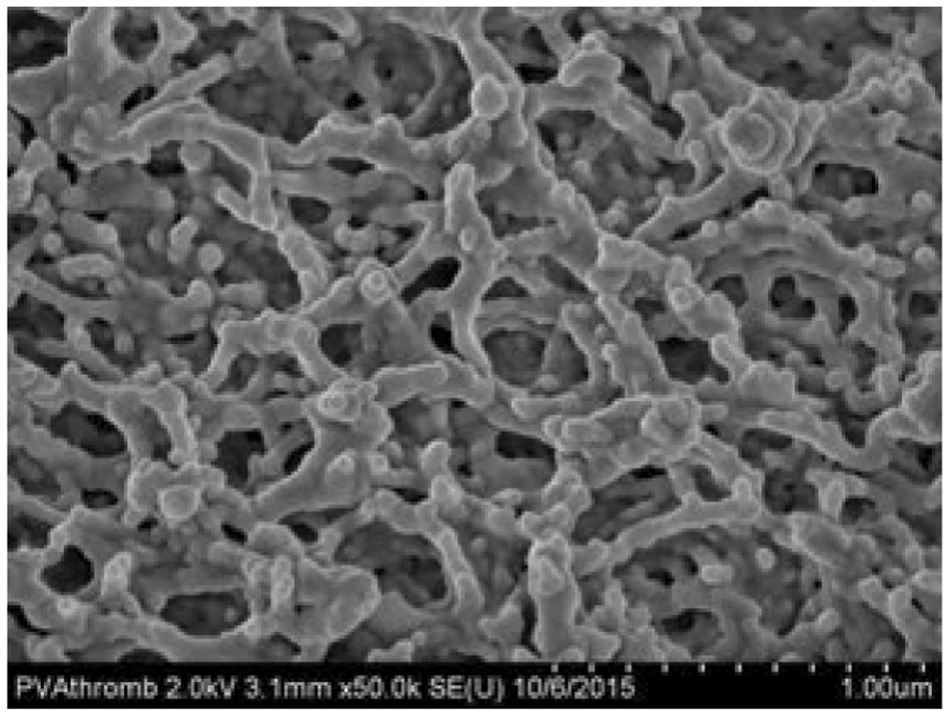
SEM image of HMB surface, showing the surface morphology

**Fig. 3:**
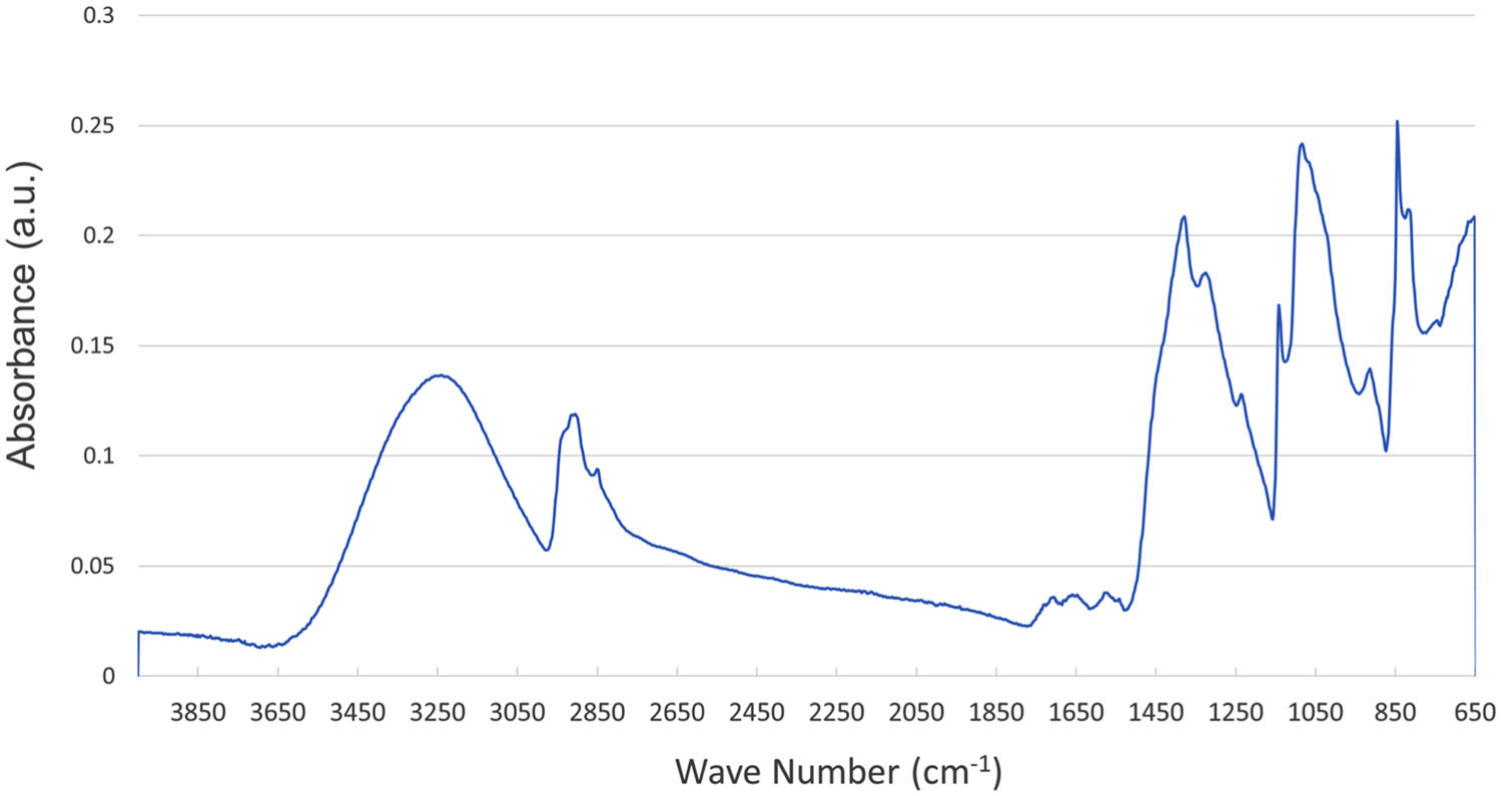
FTIR spectrum of absorption for HBM

Hydrophobic catheter materials cause thrombosis [7–9], leading to catheter-related complications including thrombotic catheter occlusion [18, 19]. HBM catheter material is extremely hydrophilic, with high wettability compared to the TPU control [15]. In a dehydrated state, the contact angle of 2ul droplets of water for PICCs made of HBM is 17 ± 6 compared to 93 ± 7 for TPU PICCs (Fig. 1) [15]. In a hydrated state, the HBM material becomes completely wetting (superhydrophilic), while the TPU contact angle decreases slightly [15]. The hydrophilicity of HBM is likely due to long hydrophilic chains throughout the composite that extend through the surface (Fig. 2). FTIR spectrum of HBM (Fig. 3) is most similar to the PVA base, but specific information about the chemical composition of HBM are trade secrets of AVI. Nonetheless, the characteristics of FTIR analysis of unmodified PVA found literature [20] are still applicable, such as the presence of a large peak around 3200 cm^−1^. This peak is linked to the stretching of O–H from the intramolecular and intermolecular hydrogen bonds [20]. The peak at 2840 cm^−1^ is related to the symmetric vibrational of C–H from alkyl groups and peak at 2920 cm^−1^ is related to the antisymmetric stretching vibrational of C–H from alkyl groups [20]. The peaks between 1750 and 1735 cm^−1^ are potentially related to the stretching C=O and C–O from the acetate group remaining from PVA [21].

All hydrogels are hydrophilic based on the nature of water absorbing into the hydrogel scaffold, but the mechanical properties vary greatly based on the type of cross-links and cross-link density [22]. The difference that makes HBM suited for vascular access applications is likely due to a combination of factors like molecular weight of components, hydrolysis of the PVA base, heat treatment, and hydrogen bonding, leading to strength and durability that most hydrogels do not possess. Young’s modulus exhibited by HBM is between 20 and 40 MPa at equilibrium water content (in vivo conditions) [15], meeting the specifications required for PICCs, including sustaining power-injection at a maximum flow rate for 11.8 cP CT contrast media of 3.5 ml/s with an average maximum catheter pressure of 910 kPa [23]. The solvation temperature of HMB is approximately 60–100 °C, and the onset of thermal degradation occurs at about 250–300 °C. Again, a TGA analysis of unmodified PVA will be considered a close approximation to that of HBM: a 10% weight loss at 295 °C, an inflection around 351 °C corresponding to a decomposition of the side chain of the PVA, and a second inflection point around 423 °C corresponding to decomposition of the main chain of PVA [20]. HyrdroPICC (AVI), constructed of HBM, is a US FDA-cleared medical device that is substantially equivalent to predicate devices and passed all required testing [13]. The antithrombotic properties of HBM are evidenced by in vitro and non-clinical in vivo testing described in literature.

**Fig. 4:**
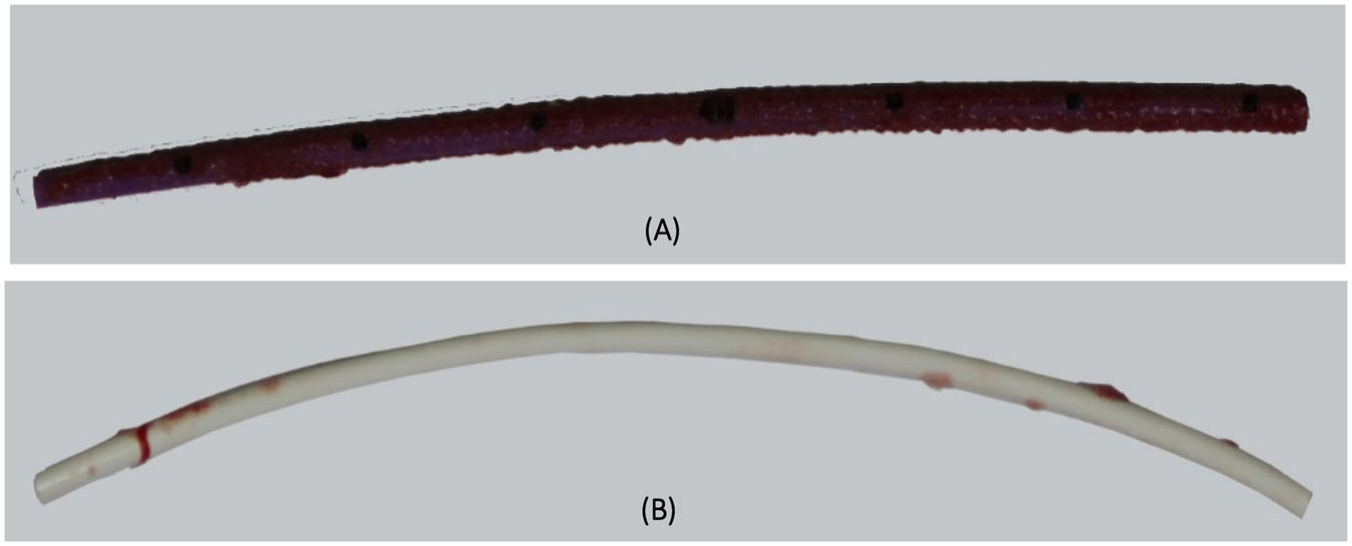
Photographic images of **A** TPU PICC and **B** HBM PICC after 120 min of testing in the in vitro blood loop model

An in vitro study of thrombus accumulation and platelet adhesion was conducted on HBM PICCs using a validated blood flow loop model (Thrombodyne, Salt Lake City, UT, USA). Fresh bovine blood was collected, and the platelets were radio-labeled with Indium-111, and samples of HydroPICC and 2 comparator PICCs, were run independently in identical closed loop systems for approximately 120 min, using the same animal for all 3 systems for each run. Thrombus accumulation was measured by radio-labeled platelet count and then normalized to the TPU control count, and HydroPICC showed up to an average of 97% reduction in platelet adhesion as compared to conventional TPU catheters, a dramatic difference (Fig. 4) [15]. While only a semi-quantitative study, the results demonstrate potent thromboresistance. An in vivo study of HBM using an ovine jugular model tested 7 HyrdroPICC devices that were implanted for 14 days (*n* = 3) and 28 days (*n* = 4). The explanted devices were evaluated for thrombus accumulation qualitatively and results confirmed the minimal seen in vitro [24].

Available clinical evidence is limited to one retrospective comparative clinical trial on MCs constructed of HBM versus a polyurethane base, conducted on 204 patient records that showed a significant reduction of catheter failures by 20.0%, including a 13% reduction in occlusions [25]. The existing research supports that increased hydrophilicity, reduces platelet adhesion, thrombus accumulation, and related complications.

The results of this study are consistent with the hypothesis that the high concentration of water at the surface of HBM may act as a disguise from the immune system, preventing thrombotic catheter occlusions from forming. Occlusion rates of PICCs reported in literature are between 7.4 and 35% [4, 18, 26], in line with this study’s finding of 21.3% for the control. Limitations of this study include the small sample size, and the general lack of controls from the nature of retrospective clinical research, e.g., standardized protocols for follow-up care and maintenance of the PICCs. Larger, prospective, randomized, controlled clinical studies are needed to understand the full risk/benefit profile of HBM in vascular access.

## Conclusion

Catheters constructed of HBM have inherently low interfacial tension associated with their hydrophilicity, and resist thrombus formation. This study examined if HBM-constructed PICCs could improve the rate of catheter occlusions versus the standard of care, a clinical manifestation of catheter-related thrombosis. The study showed that HBM technology represents a potential solution to reduce catheter-related thrombosis by fabricating PICCs from an inherently non-thrombogenic, hydrophilic, bulk hydrogel.

## Data Availability

Study data are available upon request from the corresponding author.
